# A contribution to the inventory of marine amphipod species from Italian waters based on unpublished sources and FAIR principles

**DOI:** 10.3897/BDJ.14.e189256

**Published:** 2026-04-30

**Authors:** Antonina Badalucco, Rocco Auriemma, Paolo Balistreri, Mariella Baratti, Andrea Bonifazi, Gioele Capillo, Roberta Cimmaruta, Isabella Coccia, Antonella D'Amore, Andrea Desiderato, Claudio D'Iglio, Daniele Grech, Davide Iaciofano, Loretta Lattanzi, Marco Lezzi, Monica Lionello, Eleonora Macaluso, Emanuele Mancini, Caterina Martino, Veronica Marusso, Maria Mercurio, Serena Mucciolo, Ermelinda Prato, Martina Pulieri, Patrizia Puthod, Maria Beatrice Scipione, Tommaso Scirocco, Benedetto Sirchia, Antonietta Specchiulli, Monica Targusi, Benedetta Trabucco, Andrea Vannucci, Ilaria Rosati, Sabrina Lo Brutto

**Affiliations:** 1 Department of Earth and Marine Science, University of Palermo, Palermo, Italy Department of Earth and Marine Science, University of Palermo Palermo Italy; 2 National Biodiversity Future Center (NBFC), Palermo, Italy National Biodiversity Future Center (NBFC) Palermo Italy; 3 National Institute of Oceanography and Applied Geophysics - OGS, Trieste, Italy National Institute of Oceanography and Applied Geophysics - OGS Trieste Italy; 4 ARPA Sicilia - Agenzia Regionale per la Protezione dell'Ambiente, Palermo, Italy ARPA Sicilia - Agenzia Regionale per la Protezione dell'Ambiente Palermo Italy; 5 National Research Council (CNR), Research Institute on Terrestrial Ecosystems (IRET), Sesto Fiorentino, Florence, Italy National Research Council (CNR), Research Institute on Terrestrial Ecosystems (IRET), Sesto Fiorentino Florence Italy; 6 ARPA Lazio, Dipartimento Stato dell’Ambiente, Rome, Italy ARPA Lazio, Dipartimento Stato dell’Ambiente Rome Italy; 7 Sea in Health and Life Srl, c/o Department of Chemical, Biological, Pharmaceutical and Environmental Sciences, Messina, Italy Sea in Health and Life Srl, c/o Department of Chemical, Biological, Pharmaceutical and Environmental Sciences Messina Italy; 8 Department of Chemical, Biological, Pharmaceutical and Environmental Sciences, University of Messina, Messina, Italy Department of Chemical, Biological, Pharmaceutical and Environmental Sciences, University of Messina Messina Italy; 9 Department of Ecological and Biological Sciences, University of Tuscia, Viterbo, Italy Department of Ecological and Biological Sciences, University of Tuscia Viterbo Italy; 10 Department of Biosciences, Biotechnologies and Environment, University of Bari Aldo Moro, Bari, Italy Department of Biosciences, Biotechnologies and Environment, University of Bari Aldo Moro Bari Italy; 11 Department of Biological, Geological and Environmental Sciences (BIGEA), University of Bologna, Bologna, Italy Department of Biological, Geological and Environmental Sciences (BIGEA), University of Bologna Bologna Italy; 12 National Research Council (CNR), Institute for Marine Biological Resources and Biotechnology (IRBIM), Lesina, Italy National Research Council (CNR), Institute for Marine Biological Resources and Biotechnology (IRBIM) Lesina Italy; 13 Department of Invertebrate Zoology and Hydrobiology, University of Lodz, Lodz, Poland Department of Invertebrate Zoology and Hydrobiology, University of Lodz Lodz Poland; 14 International Marine Centre (IMC), Oristano, Italy International Marine Centre (IMC) Oristano Italy; 15 Via Rodolfo Lanciani 1 00019, Tivoli (RM), Italy Via Rodolfo Lanciani 1 00019 Tivoli (RM) Italy; 16 ARPAE Emilia-Romagna - Daphne Oceanographic Structure, Cesenatico (FC), Italy ARPAE Emilia-Romagna - Daphne Oceanographic Structure Cesenatico (FC) Italy; 17 ARPA Veneto - Regional Department of Environmental Quality, Venice, Italy ARPA Veneto - Regional Department of Environmental Quality Venice Italy; 18 Department DiSTeBA, University of Salento, Lecce, Italy Department DiSTeBA, University of Salento Lecce Italy; 19 ISPRA, Italian Institute for Environmental Protection and Research, Rome, Italy ISPRA, Italian Institute for Environmental Protection and Research Rome Italy; 20 National Research Council (CNR), Water Research Institute (IRSA), Taranto, Italy National Research Council (CNR), Water Research Institute (IRSA) Taranto Italy; 21 ARPAC - Agenzia Regionale per la Protezione Ambientale in Campania, Campania, Italy ARPAC - Agenzia Regionale per la Protezione Ambientale in Campania Campania Italy; 22 Stazione Zoologica Anton Dohrn, Napoli, Italy Stazione Zoologica Anton Dohrn Napoli Italy; 23 ISPRA, BIO-ACAM Department, Rome, Italy ISPRA, BIO-ACAM Department Rome Italy; 24 APLYSIA Soc. Coop. Ricerche applicate all'ecologia e alla biologia marina, Livorno, Italy APLYSIA Soc. Coop. Ricerche applicate all'ecologia e alla biologia marina Livorno Italy; 25 National Research Council (CNR), Research Institute on Terrestrial Ecosystems (IRET), Lecce, Italy National Research Council (CNR), Research Institute on Terrestrial Ecosystems (IRET) Lecce Italy; 26 LifeWatch Italy, Lecce, Italy LifeWatch Italy Lecce Italy

**Keywords:** Amphipoda, Italian waters, Mediterranean Sea, biodiversity assessment, NBFC, GBIF-IT, LifeWatch-IT

## Abstract

Species distribution data are essential for understanding biodiversity patterns, supporting conservation planning and assessing the impacts of environmental change. Amphipods represent one of the most abundant and diverse groups of marine macroinvertebrates, encompassing over 10,900 species worldwide and exhibiting a wide range of trophic and ecological roles. Owing to their sensitivity to environmental changes, amphipods are widely used as bioindicators and are of considerable interest in both basic and applied research. However, incomplete or outdated information on species distributions has often led to misidentifications and inconsistencies in literature, highlighting the need for updated and reliable checklists.

This study presents an inventory of unpublished records comprising more than 300 species collected from the Italian maritime zones of the Adriatic, Tyrrhenian and Ionian areas. The Tyrrhenian Sea exhibited the highest taxonomic richness, whereas the Adriatic Sea accounted for the greatest number of records, likely reflecting differences in sampling effort. Eleven non-indigenous species (NIS), representing 3.6% of the total, were recorded, primarily in ports, lagoons and aquaculture facilities, while 6.6% of the species were classified as cryptogenic or of uncertain origin. Sandy and mixed substrates supported the highest species richness, consistent with their greater sampling effort. Overall, this study provides an updated overview of amphipod biodiversity and distribution in Italian seas and underscores the need for systematic and continuous monitoring. According to the FAIR (Findable, Accessible, Interoperable, Reusable) principles, the dataset was published on the GBIF (Global Biodiversity Information Facility) platform.

## Introduction

Species distribution data are crucial for understanding global biodiversity patterns, identifying priority areas for conservation and predicting the impacts of environmental change (Zhao et al. 2020). Amphipods are one of the most abundant and diverse groups of macroinvertebrates in the oceans; the order Amphipoda includes more than 10,900 species belonging to 239 families (Horton et al. 2023) and is ubiquitous, from coastal shallows to the deep sea (Hughes and Ahyong 2016). They include species of different trophic and ethological categories, they act as detritivores, herbivores, suspension feeders, scavengers, parasites or predators (Costa et al. 2021, Ritter and Bourne 2024) and are an important food source for many species of birds, fish and mammals, playing a fundamental role in the ecology of marine habitats (Dauby et al. 2003, Dauvin 2024, Ritter and Bourne 2024). Amphipods are particularly sensitive to chemical and physical changes and respond relatively quickly to natural and anthropogenic stressors; therefore, they can be used as bioindicators for monitoring the marine environment (Howard and Dorjes 1972, Borja et al. 2000, Dauvin 2018, Podlesińska and Dąbrowska 2019, Maghsoudlou et al. 2020, Navarro-Barranco et al. 2020, Lo Brutto et al. 2021, Momtazi and Maghsoudlou 2022, Mancuso et al. 2023). These crustaceans represent a group of great interest for basic (e.g. Lo Brutto and Joseph (2023)) and applied zoological research (e.g. Lo Brutto et al. (2021)). However, to fully exploit the potential of amphipods in research projects, a good knowledge of species distribution is required. This information is often incomplete or completely absent (Conlan 1994, Arfianti et al. 2018); on several occasions, the lack of distribution data has led to incorrect identifications, generating a real "domino effect" of misidentifications in subsequent literature (e.g. Lo Brutto and Iaciofano (2018), Lo Brutto and Joseph (2023)). In this context, the updating of species checklists can be considered a fundamental tool for better understanding the spatial and temporal changes that biodiversity may undergo.

An exhaustive study on the geographical distribution and ecology of the benthic amphipods of the Mediterranean Sea was carried out by Ruffo between 1982 and 1998 (Ruffo 1982, Ruffo 1989, Ruffo 1993, Ruffo 1998), mainly aimed at the taxonomic identification of species. It included around 450 species (Ruffo 1982, Ruffo 1989, Ruffo 1993, Ruffo 1998), while information on planktonic amphipods came from only a few articles (Christodoulou et al. 2013). Stephensen (Stephensen 1918, Stephensen 1924, Stephensen 1925) was the first to give a complete list of the Mediterranean pelagic amphipods, after having examined material collected during the Danish oceanographic expedition and assigned it to 80 different species. Since then, very little information has been added regarding planktonic amphipods in the Mediterranean Sea, with only a limited number of studies (e.g Bakalem and Dauvin (1995), Vinogradov et al. (1996), Zelickman (2005)). Subsequently, in 2010, Ruffo published the first Italian checklist of marine amphipods that included 457 species (388 benthic and 69 pelagic species) (Ruffo 2010).

Italy plays a central role in the biodiversity assessment of the Mediterranean Sea, as recently demonstrated by the evaluation of amphipod distribution; Badalucco et al. (Badalucco et al. 2024) showed that 77% of the species inhabiting the Mediterranean Sea are present in the Italian waters. Notwithstanding, since 2010, the Italian checklist has not been revised and gaps in information on the biodiversity and distribution of these crustaceans needed to be filled. In light of such a task, this study aimed to provide an inventory of unpublished amphipod records in Italian seas, based on data collected by various public and private research institutions. In addition to species distribution, the study includes relevant information on non-indigenous species (NIS). Increased global connectivity and human mobility have, in fact, increased the geographical spread of many taxa, particularly small organisms associated with biofouling such as amphipods (Carlton 1992, Ros et al. 2013), whose predation and competition with native species represent the most significant negative consequences known to date (Berezina 2007, Berezina et al. 2011, Meßner and Zettler 2021). At the same time, information was collected on the sampling substrates, as the structural complexity and composition of the substrate play a key role in determining amphipod assemblage (Martínez-Laiz et al. 2018, Iaciofano et al. 2024).

## Material and methods

The study area comprised the Italian territorial waters, which were subdivided into three areas: the Tyrrhenian, the Ionian (which here included the Sicily Channel) and the Adriatic Sea.

The dataset was built under a wide collaboration. Sampling was carried out by ISPRA (Istituto Superiore per la Protezione e la Ricerca Ambientale), regional ARPAs (Campania; Emilia-Romagna; Lazio; Sicily and Veneto), Universities (University of Palermo; University of Bari Aldo Moro; University of Messina; University of Salento; University of Tuscia; University of Bologna; University of Łódź), OGS (Istituto Nazionale di Oceanografia e di Geofisica Sperimentale), CNR (Consiglio Nazionale delle Ricerche) SZN (Stazione Zoologica Anton Dohrn) and IMC Foundation and private entities (Aplysia). The sampling procedures showed a great diversity, influenced by the specific needs of each research institution. They were summarised as follows: ARPA followed the guidelines established by the Marine Strategy Framework Directive (MSFD-2008/56/EC) and the Water Framework Directive 2000/60/EC (WFD-Water Framework Directive). Other institutions (public and private) adopted different sampling methodologies, which either followed a well-defined sampling design or were sporadic.

Sampling locations were mapped to visualise their spatial distribution across the Italian seas by projecting georeferenced records on to a national coastline map and displaying sampling points coloured according to the corresponding marine area (Adriatic, Tyrrhenian or Ionian Seas).

An examination of the data was conducted and barplots were produced to compare the number of records and the number of species amongst the marine areas considered. In addition, a Venn diagram was used to visualise species overlap and exclusivity amongst these areas. Records relating to identifications at the genus level were excluded from these analyses. Horizontal bar charts were built to represent the 15 families and 15 genera with the highest number of records.

Subsequent analyses focused on non-indigenous species (NIS). To describe their spatial distribution and identify areas of highest concentration along the Italian coast, a heatmap was generated to represent total abundances. Georeferenced records were aggregated into a regular spatial grid with cells of 0.4° × 0.4° (latitude x longitude). For each cell, cumulative abundance was calculated as the sum of individuals across all records; when only presence data were available, a value of 1 was assigned to ensure inclusion in the analysis. Spatial density gradients were visualised using a sequential colour scale ranging from orange to black.

To provide a detailed overview of species distribution in relation to substrates, a pie chart was generated, followed by a barplot to quantify the variety of substrates present in each of the three seas considered. The composition of marine substrates was examined using a stacked barplot, which showed the number of observations for each substrate type within each area.

Records with missing data relating to the sampling year or substrate type, as well as observations referring to the “pelagic water column”, were excluded from the statistical analyses and specific graphs for these variables.

All descriptive analyses were performed using R version 4.4.2 (R Core Team 2025).

### Project description

NBFC is the first National Research and Innovation Centre dedicated to biodiversity, funded by the MUR through European Union funds NextGenerationEU. It is a coordinating structure that unites and enhances research efforts, while simultaneously making knowledge and technologies accessible to diverse actors working in the territory. There are more than 2000 researchers from research centres, universities and companies working within the Centre implementing basic, applied and innovation research actions dedicated to Mediterranean biodiversity to generate value for the country. The objective of this project is to identify suitable strategies for monitoring, conserving, restoring and valorising the biodiversity of species and habitats in different Italian regions. The Centre provides scientific knowledge and technological innovation that make it possible to face biodiversity loss, support ecosystem resilience, monitor endangered species and restore disturbed biological communities, contributing to the goal of protecting 30% of Italy's territory by 2030, as required by the European Union.

## The GBIF occurrence dataset

Based on the importance of open access to primary scientific knowledge, the dataset herein introduced was made available online as it was uploaded to the GBIF platform (Badalucco and Lo Brutto 2025). The analysed dataset integrates sampling data on amphipods collected by public and private research institutes, providing an updated inventory of their distribution in Italian marine waters between the early 1980s and 2025. Each record is associated with taxonomic information (i.e. kingdom, phylum, class, order, family, genus and species) and details of the sampling event, including record identifier, date, locality, geographical coordinates and sampling protocol (Table 1). All species were found in a bathymetric range from 0 to 110 m. The study area is geographically delimited by the following boundary coordinates: from the northernmost point (45.742385 N, 13.269923 E) to the southernmost point (37.03257 N, 14.25354 E) and from the westernmost point (40.563565 N, 8.313818 E) to the easternmost point (40.106794 N, 18.522000 E). Geographical information has been organised by dividing the records into the three main areas: Tyrrhenian, Ionian (including the Sicily Channel) and Adriatic. The boundaries have been set at Mazara del Vallo, between the Tyrrhenian and Ionian Seas and Otranto, between the Ionian and Adriatic Seas, using the “waterBody” column to assign each record. In order to characterise the habitats of the studied species, data on sampling substrates were collected. These categories were standardised according to the Habitat types of the European Nature Information System (EUNIS). The substrates were standardised according to the EUNIS classification (2021/2022 version), with the exception of artificial substrates, for which the 2012 version was adopted (Table 2). Information about whether organisms have been introduced to a given place and time through the direct or indirect activity of modern humans was provided using the Establishment Means Controlled Vocabulary. The taxonomic information was updated in line with the World Amphipoda Database (Horton et al. 2025).

## Results

**Figure 1.**
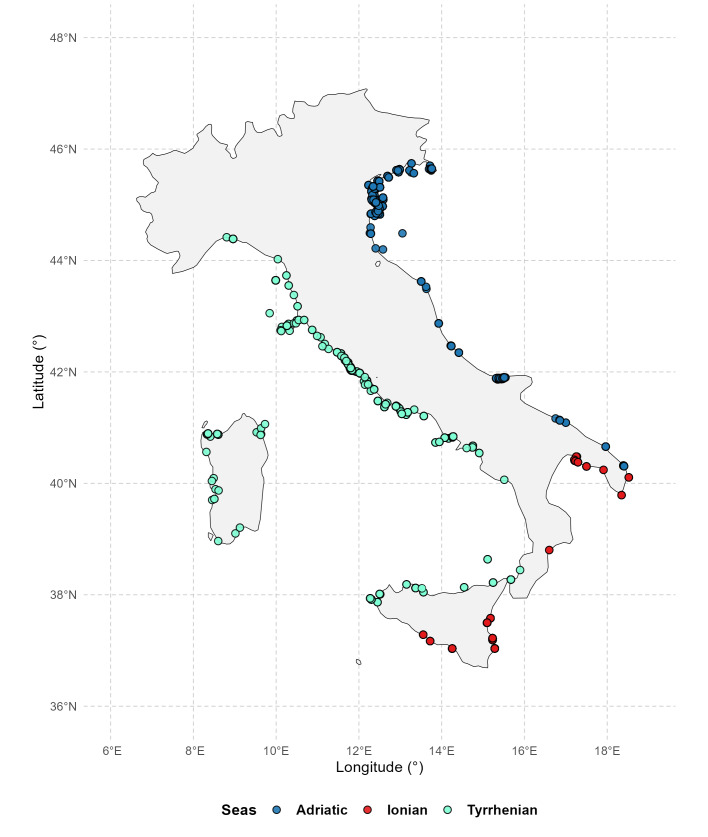
Map showing the distribution of the georeferenced species records. Points were differentiated by colours, based on the sea upon which they lie: Tyrrhenian Sea (light blue), Ionian Sea (red) and Adriatic Sea (blue).

A total of 4344 amphipod records were detected, distributed in 379 geographic coordinates (Fig. 1). A total of 302 species belonging to 127 genera, 51 families and four suborders were recorded.

**Figure 2.**
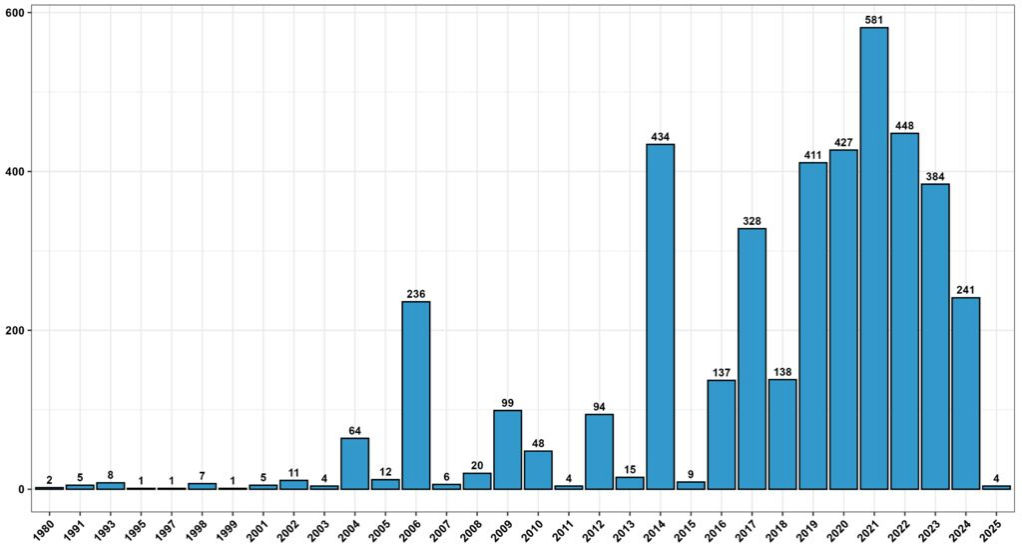
Graph showing the distribution of records through the years from 1980 to 2025.

The temporal distribution of the data revealed a marked increase in records over time (Fig. 2). The number of records remained consistently low from 1980 to the early 2000s, then fluctuated until 2013, with an initial significant peak in 2006 (236 records). From 2014 onwards, a substantial increase was observed, reaching a record high in 2021 (581 records) and maintaining elevated values throughout the 2017-2023 period. The increase is attributable to the launch of monitoring activities under the Marine Strategy framework in Italy, which entails the systematic collection of environmental data to assess the ecological status of marine waters. Consequently, the dataset is dominated by records collected in the last decade, while the lower numbers recorded in 2024 and 2025 reflect partial data and are still being processed.

**Figure 3.**
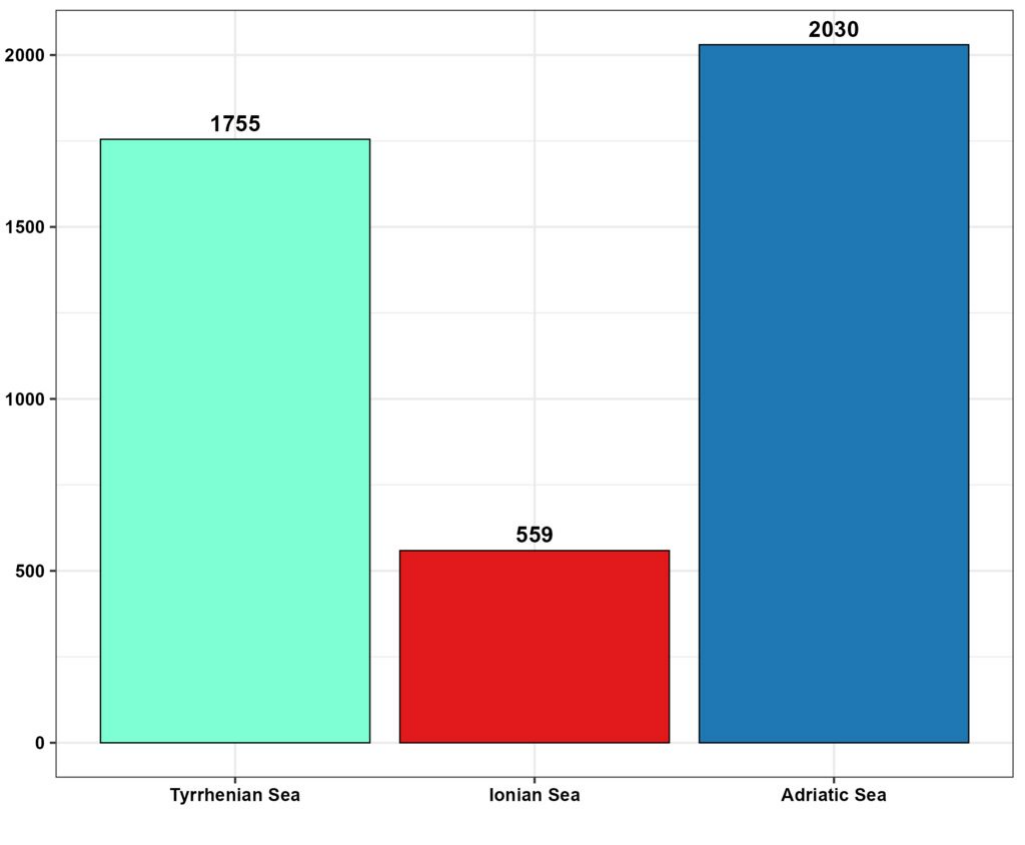
Number of records per sea.

**Figure 4.**
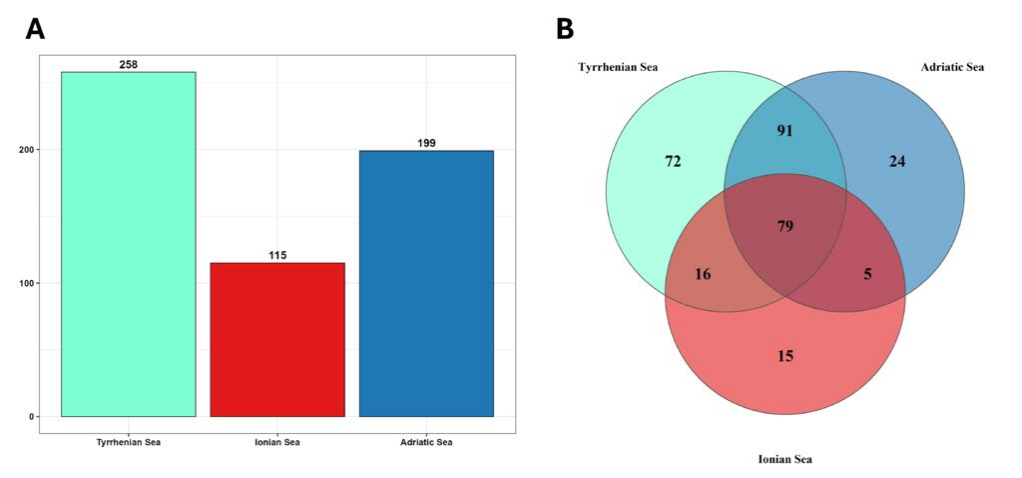
**A** Number of species per sea; **B** Venn diagram illustrating the distribution and shared amphipod species across the three areas, Tyrrhenian Sea (light blue), Ionian Sea (red), Adriatic Sea (blue).

A notable difference emerged from the number of records listed for the different seas: 2030 records were counted for the Adriatic, 1755 for the Tyrrhenian and 559 for the Ionian area (Fig. 3). The distribution of amphipods revealed that the Tyrrhenian Sea hosted the largest number of taxa across all taxonomic ranks (49 families, 109 genera and 258 species); followed by the Adriatic Sea with 40 families, 90 genera and 199 species and the Ionian Sea with 32 families, 65 genera and 115 species (Fig. 4, A). The occurrence data showed that 79 species were common to all three seas (Tyrrhenian, Adriatic and Ionian). Ninety-one species were shared between the Tyrrhenian and Adriatic Seas, 16 between the Tyrrhenian and Ionian Seas and five between the Adriatic and Ionian Seas. Seventy-two species were exclusive to the Tyrrhenian, 15 to the Ionian and 24 to the Adriatic (Fig. 4, B).

**Figure 5.**
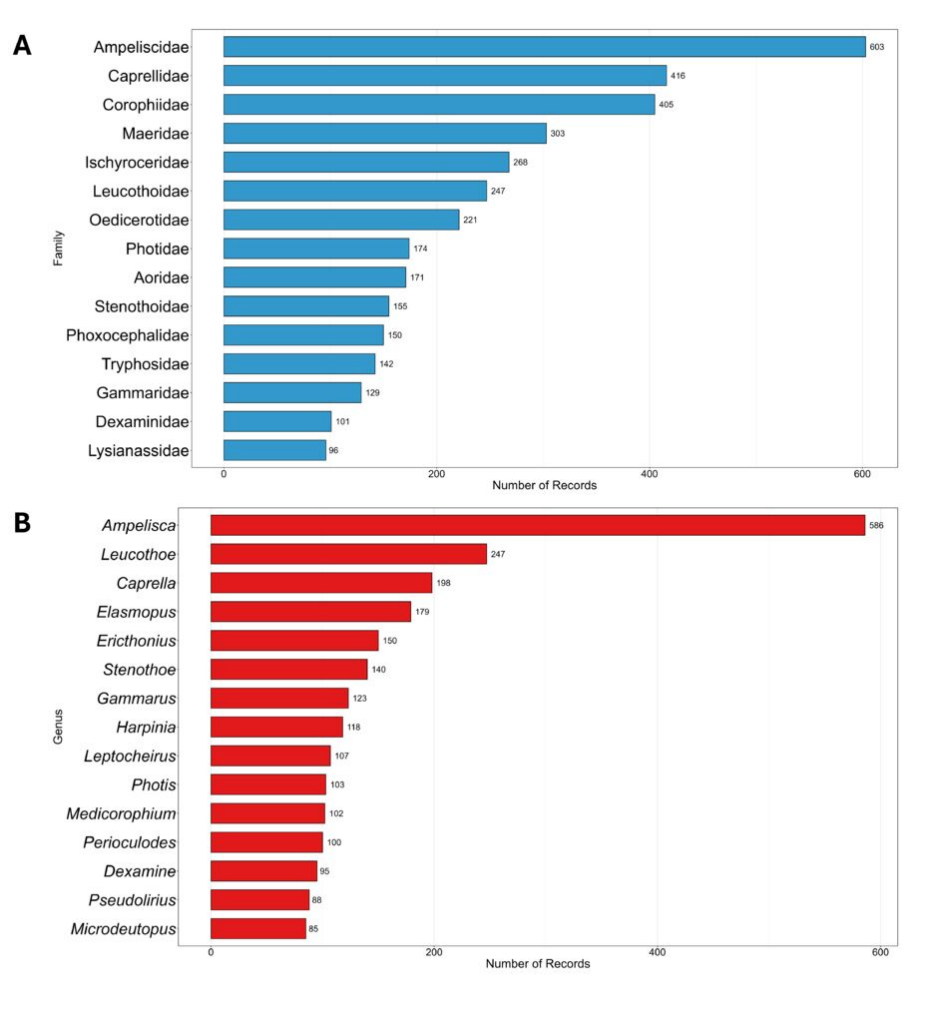
Barplot showing. **A** the 15 most frequently sampled families; **B** the 15 most frequently sampled genera.

From a taxonomic point of view, the family Ampeliscidae Krøyer, 1842 and the genus *Ampelisca* Krøyer, 1842 were the most frequently sampled, representing 13.9% and 13.5% of total records, respectively (Fig. 5, A-B). The most commonly sampled species, in terms of number of records, were *Ampelisca typica* (Spence Bate, 1857) (152 records); *Elasmopus rapax* A. Costa, 1853 (140 records); *Ampelisca intermedia* Bellan-Santini & Diviacco, 1990 (104 records); *Photis longicaudata* (Spence Bate & Westwood, 1862) (103 records) and *Pseudolirius kroyeri* (Haller, 1879) (88 records). The most abundant species in terms of number of individuals per single record were *Corophium orientale* Schellenberg, 1928 (3971 individuals in a single record); *Monocorophium insidiosum* (Crawford, 1937) (3358); *Ampelisca intermedia* Bellan-Santini & Diviacco, 1990 (2770); *Gammarus insensibilis* Stock, 1966 (2745) and *Pseudolirius kroyeri* (Haller, 1879) (2137). Species considered rare (in terms of the lowest record numbers) were detected with the following frequencies: 57 species occurred with only one record; 34 species with two records; and 30 species with three records.

**Figure 6.**
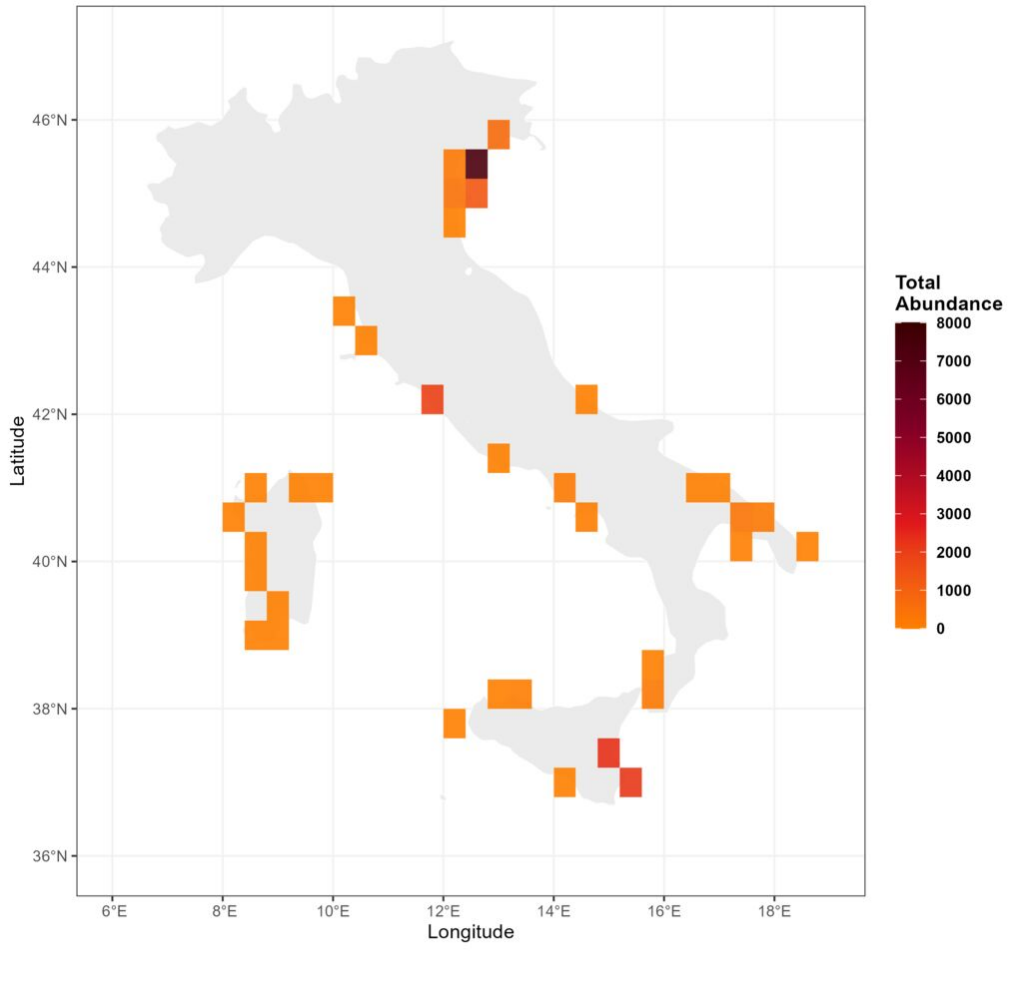
Heatmap of the total abundance of the eleven NIS along the Italian coastline (*A. valida*; *C. scaura*; *E. pectenicrus*; *E. didymus*; *G. japonica*; *J. marmorata*; *J. slatteryi*; *L. baconi*; *P. cypris*; *P. littoralis*; *S. georgiana*). The colour intensity (from orange to black) indicates the cumulative density of individuals within grid cells with a resolution of 0.4° × 0.4° (Lat x Long). The values represent the sum of the abundances recorded for each geographical sector.

Eleven species were non-indigenous (NIS) for the Mediterranean Sea: *Ampithoe valida* S.I. Smith, 1873; *Caprella scaura* Templeton, 1836; *Elasmopus pectenicrus* (Spence Bate, 1863); *Ericthonius didymus* Krapp-Schickel, 2013; *Grandidierella japonica* Stephensen, 1938; *Jassa marmorata* Holmes, 1905; *Jassa slatteryi* Conlan, 1990; *Laticorophium baconi* (Shoemaker, 1934); *Parametopella cypris* Holmes, 1905; *Ptilohyale littoralis* (Stimpson, 1853) and *Stenothoe georgiana* Bynum & Fox, 1977 (Fig. 6).

Specifically, in the northern Adriatic Sea, twenty specimens of *A. valida* and twenty of *P. littoralis* were sampled, both found in oyster farms; in the same area, 1169 specimens of *G. japonica* and 1413 of *P. cypris* were collected. *E. didymus* was recorded with 469 specimens in both the Tyrrhenian Sea (Civitavecchia) and the northern Adriatic Sea, while 2391 specimens of *L. baconi* and 3882 of *S. georgiana* were sampled in all three Italian seas. *Laticorophium baconi* was a new record for the port of Palermo (southern Italy).

Four species were already present as NIS in Ruffo's checklist (Ruffo 2010): *C. scaura*; *E. pectenicrus*; *J. slatteryi* and *J. marmorata*. Here, their presence in Italian waters was confirmed. *C. scaura* was recorded with 2298 specimens in the Tyrrhenian, Ionian and Adriatic Sea; *E. pectenicrus* with 187 specimens in the Tyrrhenian and Ionian; *J. slatteryi* with 3905 specimens in the Tyrrhenian, Ionian and Adriatic; and *J. marmorata* with 1508 specimens in the Tyrrhenian, Ionian and Adriatic Sea.

According to literature, twenty species have an uncertain or cryptogenic status and included: *Apocorophium acutum* (Chevreux, 1908); *Apohyale perieri* (Lucas, 1846); *Caprella danilevskii* Czerniavsky, 1868; *Caprella dilatata* Krøyer, 1843; *Caprella equilibra* Say, 1818; *Caprella penantis* Leach, 1814; *Cymadusa filosa* Savigny, 1816; *Elasmopus rapax* A. Costa, 1853; *Ericthonius brasiliensis* (Dana, 1853); *Ericthonius difformis* H. Milne Edwards, 1830; *Ericthonius punctatus* (Spence Bate, 1857); *Hamimaera hamigera* (Haswell, 1879); *Latigammaropsis togoensis* (Schellenberg, 1925); *Leucothoe spinicarpa* (Abildgaard, 1789); *Maera grossimana* (Montagu, 1808); *Monocorophium insidiosum* (Crawford, 1937); *Monocorophium sextonae* (Crawford, 1937); *Perioculodes aequimanus* (Kossman, 1880); *Photis longicaudata* (Spence Bate & Westwood, 1862); and *Stenothoe valida* Dana, 1853.

**Figure 7.**
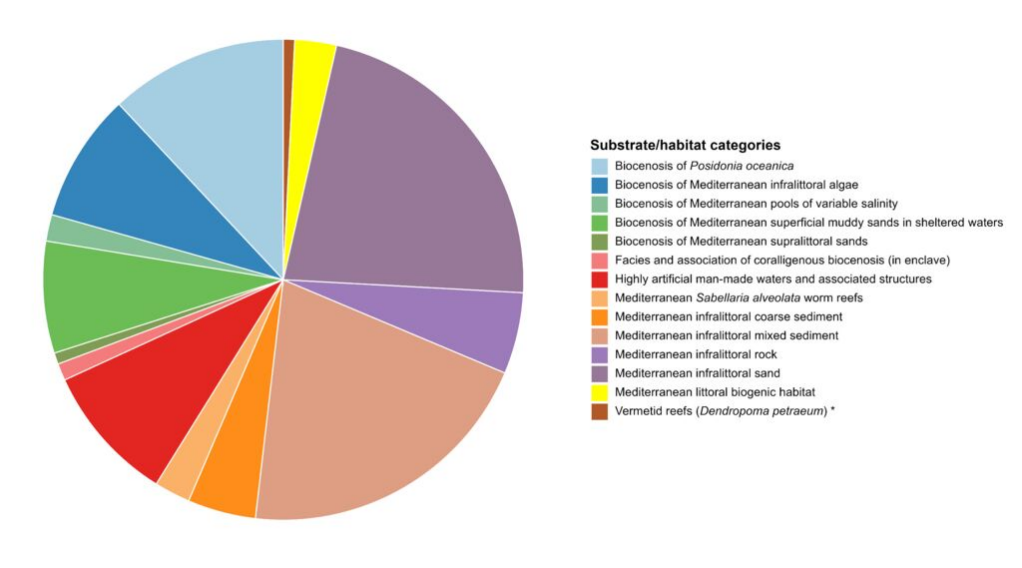
Percentage of the amphipod species per substrate/habitat categories. *The species *Dendropoma petraeum* is now accepted as *Dendropoma cristatum*.

The species were sampled from different substrates. Seventy-three species were sampled on “Highly artificial man-made waters and associated structures”; 43 on “Mediterranean infralittoral rock”; 19 on “Mediterranean *Sabellaria alveolata* worm reefs”; six on “Vermetid reefs (*Dendropoma petraeum*)”; nine on “Facies and association of coralligenous biocenosis (in enclave)”; 22 on “Mediterranean littoral biogenic habitat”; 68 on “Biocenosis of Mediterranean infralittoral algae”; 94 on “Biocenosis of *Posidonia oceanica*”; 59 on “Biocenosis of Mediterranean superficial muddy sands in sheltered waters”; 175 on “Mediterranean infralittoral sand”; 36 on “Mediterranean infralittoral coarse sediment”; and 161 on “Mediterranean infralittoral mixed sediment”. Only 14 species were found on “Biocenosis of Mediterranean pools of variable salinity” and six on “Biocenosis of Mediterranean supralittoral sands” (Fig. 7).

**Figure 8.**
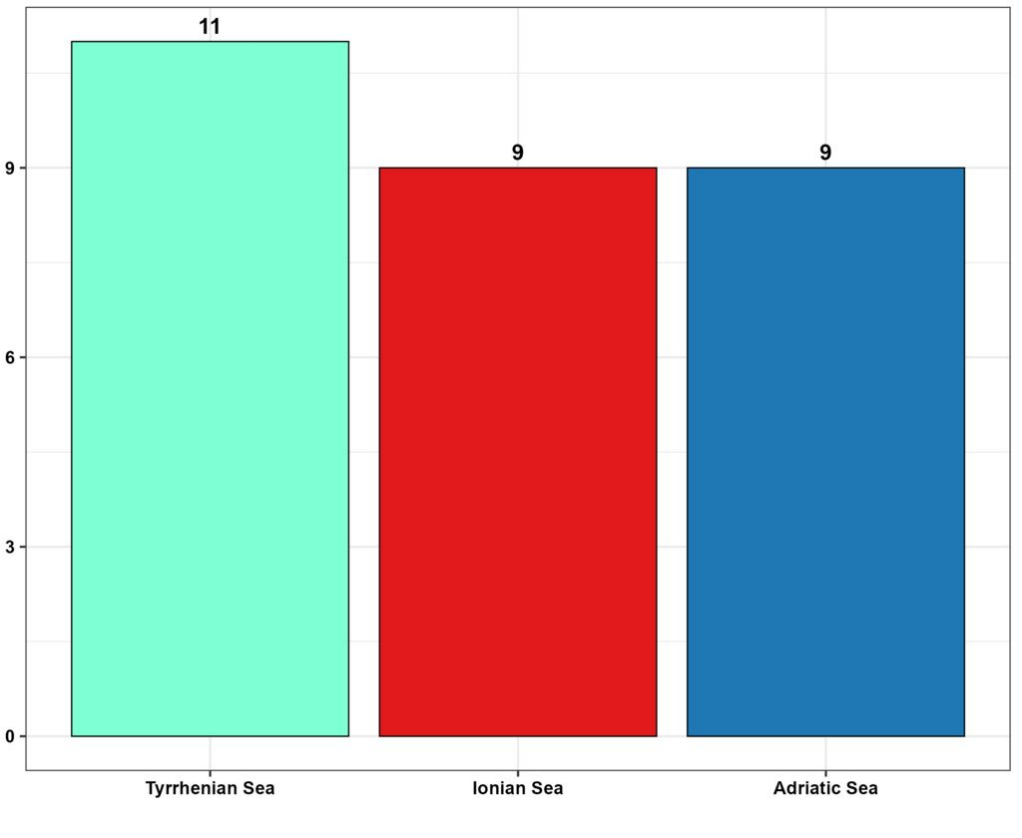
Number of investigated substrate categories per sea; Tyrrhenian Sea (light blue), Ionian Sea (red) and Adriatic Sea (blue).

**Figure 9.**
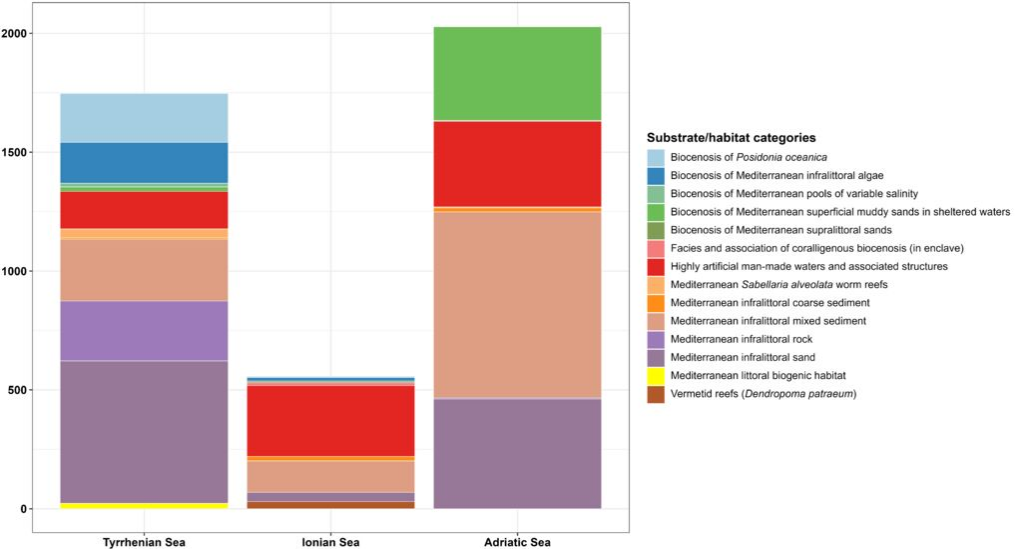
Stacked barplot showing the distribution of the number of substrate detections for each sea (Tyrrhenian, Ionian and Adriatic). *The species *Dendropoma petraeum* is now accepted as *Dendropoma cristatum*.

Samples from the Tyrrhenian Sea encompassed the highest number of substrate categories (11), whereas those from the Ionian and Adriatic Seas each included nine categories (Fig. 8). The Adriatic and Tyrrhenian Seas recorded the greatest number of detections and were dominated by soft substrates, particularly “Mediterranean infralittoral mixed sediments” and “Mediterranean infralittoral sands”. In contrast, the Ionian Sea, despite a lower overall number of records, exhibited a higher proportion of artificial substrates, mainly “Highly artificial man-made waters and associated structures” (Fig. 9).

The analysis showed that the species present in the highest number of substrate/habitat categories were *Ampithoe ramondi* Audouin, 1826 and *A. acutum* (both collected from nine substrates) followed by *C. scaura*, *Phtisica marina* Slabber, 1769, *Gammarella fucicola* (Leach, 1814), *E. rapax*, *E. punctatus* and *Quadrimaera inaequipes* (A. Costa in Hope, 1851), found in eight different habitats. Finally, *Lysianassa costae* H. Milne Edwards, 1830 was sampled in seven substrate/habitat categories.

In this study, the detected semi-terrestrial amphipods were *Orchestia montagui* Audouin, 1826 (324 individuals), *Orchestia mediterranea* A. Costa, 1853 (249 individuals), *Cryptorchestia garbinii* Ruffo, Tarocco & Latella, 2014 (70 individuals), *Platorchestia platensis* (Krøyer, 1845) (89 individuals), *Speziorchestia stephenseni* (Cecchini, 1928) (268 individuals) and *Talitrus platycheles* Guérin, 1832 (5 individuals). From the Ionian Sea, the study reported *O. montagui* and *O. mediterranea*, together with new records of *C. garbinii* and *S. stephenseni*. The presence of *P. platensis* was confirmed in both the Ionian Sea and the Adriatic Sea. Finally, for the southern Adriatic, a new record of *T. platycheles* was reported. These species were associated with the substrate “Biocenosis of Mediterranean supralittoral sands”.

Of the total records, only three species were planktonic: *Vibilia gibbosa* Bovallius, 1887 (2 individuals), *Phrosina semilunata* Risso, 1822 (3 individuals) and *Anchylomera blossevillei* H. Milne Edwards, 1830 (57 individuals). Two belonging to the family Phrosinidae: *P. semilunata* and *A. blossevillei*, were both found in the Ionian Sea. *Vibilia gibbosa* is a new record for Italy, found in the port of Naples. These three species have been included in the category “Pelagic water column”.

The whole dataset can be downloaded from the GBIF platform https://doi.org/10.15468/2u8gud (Badalucco and Lo Brutto 2025).

## Discussion

The Mediterranean Sea represents one of the major global hotspots of amphipod diversity, with more than 500 species recorded to date (Christodoulou et al. 2013, Badalucco et al. 2024) . Although numerous studies have investigated amphipod fauna in Italian waters, no updated national species checklist has been available since the last inventory published in 2010 (Ruffo 2010). The regular updating of faunal checklists is a fundamental step towards improving the understanding of marine biodiversity patterns at both national and Mediterranean scales (Bakalem et al. 2021, Fišer et al. 2021, Badalucco et al. 2024, Azzurro et al. 2025). Within this framework, amphipods represent a particularly informative taxonomic group, as they play a key ecological role in marine ecosystems by linking primary producers to higher trophic levels and contributing substantially to nutrient cycling (Plicanti et al. 2017, Lo Brutto et al. 2021, Ritter and Bourne 2024).

This study provides a comprehensive and updated overview of amphipod diversity and distribution in Italian marine waters, reporting unpublished records for 302 species distributed across the national territory. Marked differences in the number of records were observed amongst the three major Italian marine sectors, with higher values in the Adriatic and Tyrrhenian Seas compared to the Ionian Sea. These patterns likely reflect a combination of habitat heterogeneity and irregular sampling effort. The Adriatic Sea is historically amongst the most intensively studied Mediterranean basins (Servello et al. 2019), owing to its relatively shallow depths and high accessibility, factors that can account for its high number of records. On the other side, the lower number of records from the Ionian Sea is more plausibly attributable to limited sampling coverage rather than to a true deficit in species richness.

It is noteworthy that, despite the Adriatic Sea exhibiting the highest number of records, the Tyrrhenian Sea showed the greatest taxonomic richness across all hierarchical taxonomic ranks, i.e. families, genera and species. This finding suggests that the Tyrrhenian Sea may represent a hotspot of amphipod diversity, maybe driven by its pronounced bathymetric variability and wide range of available habitats.

Italy is amongst the most active European countries in the compilation of faunal checklists (Bologna et al. 2022). To date, national checklists have been published for a wide array of terrestrial, freshwater and marine taxa, covering more than 27,600 species and subspecies, with additional lists currently in preparation by taxonomic specialists (https://www.lifewatchitaly.eu/iniziative/checklist-fauna-italia-it/checklist-table/). Recent updates include molluscs (Renda et al. 2022), rotifers (Fontaneto et al. 2022), mammals (Loy et al. 2019) and several insect groups, such as Odonata (La Porta et al. 2023), Heteroptera (Cianferoni et al. 2024) and Formicidae (Schifani 2022). Moreover, checklists derived from museum collections, such as those focusing on mammal and crustacean collections (Lo Brutto et al. 2023, Pipitone et al. 2023), are of promising importance for biodiversity conservation, as they document both historical and contemporary species occurrences and enable the assessment of biodiversity changes in response to anthropogenic pressures, including habitat loss and degradation.

Comparable national or regional amphipod inventories have been compiled in several other Mediterranean and European countries. In France, more than 300 amphipod species have been reported, including a substantial proportion of planktonic taxa (Dauvin 2022, Bachelet et al. 2003). Approximately 200 species, including freshwater taxa, have been recorded in Slovenia (Fišer et al. 2021). Tunisia remains comparatively understudied, with around 150 species documented (Zakhama-Sraieb et al. 2017), whereas the Algerian coast has a more comprehensive inventory, reporting 332 species (Bakalem et al. 2021). Nevertheless, the North African coastline remains insufficiently explored and requires further systematic investigation (Zakhama-Sraieb et al. 2017, Fersi et al. 2018, Khammassi et al. 2019, Bakalem et al. 2021). In Greece, approximately 300 species have been reported, primarily from the Aegean Sea (Christodoulou et al. 2013). In contrast, relatively limited amphipod species lists are currently available for countries such as Israel and Spain (Sorbe and Galil 2002, De-La-Ossa-Carretero et al. 2010, Navarro-Barranco et al. 2023, Iaciofano et al. 2024, Iaciofano et al. 2026).

In this study, 3.64% of the recorded amphipod species were classified as non-indigenous species (NIS), while 6.62% were assigned an uncertain biogeographic origin. These findings are consistent with those reported by Marchini and Cardeccia (2017), who highlighted that, regarding amphipods, records of uncertain origin substantially exceed those of confirmed non-indigenous taxa, reflecting persistent taxonomic and biogeographic ambiguities affecting several species.

All eleven NIS identified in the present study shared a common habitat trait, having been recorded mostly in ports and marinas, aquaculture facilities or lagoonal environments. Their occurrences were primarily concentrated in the Venice Lagoon, historically recognised as the principal gateway for marine NIS introductions in Italy (Occhipinti-Ambrogi et al. 2010, Servello et al. 2019) and in the western Ionian sector (Fig. 6). Of the eleven NIS species documented in this study, maritime traffic and transport associated with shellfish farming appear to be the primary vectors of introduction. The latter represents a key surveillance area for the westward expansion of NIS originating from the eastern Mediterranean, particularly along the Sicilian coastline (Bianchi et al. 2013, Servello et al. 2019). Within this region, prominent invasion hotspots include the Gulf of Catania and the Augusta area (Siracusa), which are characterised by intense maritime traffic associated with major commercial ports, petrochemical complexes and national and NATO military infrastructures (D’Alessandro et al. 2018).

The data on non-indigenous amphipods provided a current overview of their distribution patterns, which range from spatial stability to marked geographical expansion. Several species, including *A. valida*, *P. cypris* and *P. littoralis*, exhibited distributional stability, confirming geographical patterns previously reported in literature (Desiderato et al. 2019, Langeneck et al. 2023, Ragkousis et al. 2023) and showing no evidence of expansion beyond their known ranges, which remain largely restricted to the northern Adriatic Sea.

In contrast, *C. scaura*, initially reported only from the northern Adriatic, now displays a widespread distribution along the entire Italian coastline, in agreement with recent literature (Krapp et al. 2006, Cabiddu et al. 2013, Dailianis et al. 2016, Lo Brutto et al. 2018, Langeneck et al. 2023, Ragkousis et al. 2023, Pierri et al. 2024). A similar expansion trend was observed for *J. slatteryi*, whose range, previously limited to the northern Adriatic and the Tyrrhenian Sea (Langeneck et al. 2023, Mancuso et al. 2023, Ragkousis et al. 2023), is here shown to extend to the Apulian coast and the Ionian sector of Sicily.

For *E. pectenicrus*, the data confirmed its continued presence in the Tyrrhenian Sea and the Strait of Messina (D'Alessandro et al. 2016, Mancuso et al. 2021), as well as a range extension into the Sicily Channel and the Ionian Sea; however, no new records have been reported from the Adriatic Sea since 2010. New regional records are also documented for *E. didymus*, now recorded in the Lazio and Apulia regions and for *L. baconi*, with a first record from the port of Palermo.

Finally, while *J. marmorata* and *S. georgiana* further consolidate their broad distributions along the Italian coastline (D'Alessandro et al. 2016, Fernandez-Gonzalez and Sanchez-Jerez 2017, Ulman et al. 2017, Servello et al. 2019, Ragkousis et al. 2023), with a new Ionian record for the former. *Grandidierella japonica* exhibited a more restricted pattern, characterised by a progressive spread along the Adriatic coast from Veneto to Emilia-Romagna.

It is well known that habitat selection in amphipods is mediated by multiple environmental variables and that substrate composition emerges as a determining factor in modelling their distribution (Clinton et al. 2018, Martínez-Laiz et al. 2018, Iaciofano et al. 2024). Given the importance of the substrate for amphipod ecology, this study collected and analysed the distribution of species in relation to different habitat/substrate types. The results showed a greater species richness in soft bottoms, with particular reference to “sand” and “mixed sediment” (Fig. 7). However, it should be noted that the greater diversity detected could be influenced by a major sampling effort dedicated to these substrates/habitats, which allowed for a more in-depth characterisation. As a result, this study also highlights how some types of substrates are still significantly less investigated than others.

Marine biodiversity exhibits pronounced variation across spatial and temporal scales, with the magnitude and direction of these changes strongly influenced by environmental conditions and taxon-specific responses (Lo Brutto and Iaciofano 2020, Iaciofano et al. 2024, Steger et al. 2024). However, robust quantitative assessments of such dynamics are not feasible in the absence of regularly updated and comprehensive species inventories. This study aimed to update the amphipod species list for Italian marine waters and to make these data openly available through global biodiversity platforms, such as the Global Biodiversity Information Facility (GBIF), thereby ensuring broad accessibility, visibility and reusability at an international scale. The availability of curated, open-access datasets represents a critical step towards facilitating comparative analyses, long-term monitoring and evidence-based biodiversity assessments.

Significant knowledge gaps persist for many amphipod species, owing both to the inherent difficulty of detecting and sampling certain taxa and to the limited taxonomic and ecological attention historically devoted to other groups. These limitations are further exacerbated by the near absence of targeted sampling efforts and research on planktonic amphipods, which constitute a fundamental component of amphipod biodiversity, but remain largely overlooked in contemporary studies.

An additional and often underestimated challenge concerns data standardisation. The lack of shared sampling and reporting protocols restricts the construction of reliable time series for temporal comparisons and hampers the application of statistical analyses, which are essential for advancing our understanding of amphipod distribution patterns and ecological drivers.

In conclusion, the present study represents a rare example of a coordinated good-practice initiative within the Italian scientific context. It is based on a strong collaboration amongst specialised research groups, provides a long-term and spatially extensive dataset and facilitates data integration into an interoperable, FAIR-compliant repository such as GBIF, thereby contributing to more transparent, reproducible and impactful marine biodiversity research.

Despite the remarkable biodiversity of amphipod crustaceans in the Italian waters, the full potential of genetic research is hindered by the absence of a comprehensive molecular library. The recent MEDAMP (Mediterranean and Adjacent Seas Amphipod Reference Library) inititive has been developed to address the existing knowledge gap regarding the biodiversity of amphipod crustaceans in the Mediterranean Basin. The project involves the establishment of a systematic molecular reference library. The initiative, grounded in a collaborative and open-access paradigm, centres on the standardised sequencing of cytochrome c oxidase (COI) subunit I, utilising morphologically identified specimens.

## Funding

Project funded under: (a) National Recovery and Resilience Plan (NRRP), Mission 4 Component 2 Investment 1.4, of the Italian Ministry of University and Research funded by the European Union – NextGenerationEU; Project code CN_00000033, Concession Decree No. 1034 of 17 June 2022 adopted by the Italian Ministry of University and Research, CUP D33C22000960007 and CUP B73C22000790001, Project title “National Biodiversity Future Center – NBFC”; (b) Accordo Operativo MATTM-ISPRA-ARPA del 11 Gennaio 2018 di attuazione del D.Lgs. N. 190/2010 di recepimento della Direttiva 2008/56/CE (Direttiva Quadro sulla Strategia Marina) 2018-2020; (c) Accordo Operativo MATTM-ISPRA-ARPA del 28 Gennaio 2021 di attuazione del D.Lgs. N. 190/2010 di recepimento della Direttiva 2008/56/CE (Direttiva Quadro sulla Strategia Marina) 2021-2023; (d) this study was conducted in partial fulfilment of the requirements for the PhD thesis of A. D. International PhD programme ‘Innovative technologies and sustainable use of Mediterranean Sea fishery and Biological Resources’ (www.FishMed-PhD.org); (e) IDUB project B2211001000118.07 of the University of Lodz, Poland; and (f) FFR-2025 by the University of Palermo.

## Figures and Tables

**Table 1.** Description of the data table attributes.

**Attribute name**	**Attribute definition**
institutionCode	The name (or acronym) in use by the institution having custody of the object(s) or information referred to in the record.
basisOfRecord	The specific nature of the data record.
occurrenceID	An identifier for the dwc:Occurrence (as opposed to a particular digital record of the dwc:Occurrence). In the absence of a persistent global unique identifier, construct one from a combination of identifiers in the record that will most closely make the dwc:occurrenceID globally unique.
recordedBy	A list (concatenated and separated) of names of people, groups or organisations responsible for recording the original dwc:Occurrence. The primary collector or observer, especially one who applies a personal identifier (dwc:recordNumber), should be listed first.
recordedByID	A list (concatenated and separated) of the globally unique identifier for the person, people, groups or organisations responsible for recording the original dwc:Occurrence.
organismQuantity	A number or enumeration value for the quantity of dwc:Organisms.
organismQuantityType	The type of quantification system used for the quantity of dwc:Organisms.
establishmentMeans	Statement about whether a dwc:Organism has been introduced to a given place and time through the direct or indirect activity of modern humans.
occurrenceStatus	A statement about the presence or absence of a dwc:Taxon at a dcterms:Location.
eventDate	The date-time or interval during which a dwc:Event occurred. For occurrences, this is the date-time when the dwc:Event was recorded. Not suitable for a time in a geological context.
year	The four-digit year in which the dwc:Event occurred, according to the Common Era Calendar.
habitat	A category or description of the habitat in which the dwc:Event occurred.
samplingProtocol	The names of, references to, or descriptions of the methods or protocols used during a dwc:Event.
higherGeography	A list (concatenated and separated) of geographic names less specific than the information captured in the dwc:locality term.
continent	The name of the continent in which the dcterms:Location occurs.
waterBody	The name of the water body in which the dcterms:Location occurs.
countryCode	The standard code for the country in which the dcterms:Location occurs.
stateProvince	The name of the next smaller administrative region than country (state, province, canton, department, region etc.) in which the dcterms:Location occurs.
municipality	The full, unabbreviated name of the next smaller administrative region than county (city, municipality etc.) in which the dcterms:Location occurs. Do not use this term for a nearby named place that does not contain the actual dcterms:Location.
verbatimLocality	The original textual description of the place.
decimalLatitude	The geographic latitude (in decimal degrees, using the spatial reference system given in dwc:geodeticDatum) of the geographic centre of a dcterms:Location. Positive values are north of the Equator, negative values are south of it. Legal values lie between -90 and 90, inclusive.
decimalLongitude	The geographic longitude (in decimal degrees, using the spatial reference system given in dwc:geodeticDatum) of the geographic centre of a dcterms:Location. Positive values are east of the Greenwich Meridian, negative values are west of it. Legal values lie between -180 and 180, inclusive.
acceptedNameUsageID	An identifier for the name usage (documented meaning of the name according to a source) of the currently valid (zoological) or accepted (botanical) taxon.
scientificName	The full scientific name, with authorship and date information if known. When forming part of a dwc:Identification, this should be the name in lowest level taxonomic rank that can be determined. This term should not contain identification qualifications, which should instead be supplied in the dwc:identificationQualifier term.
acceptedNameUsage	The full name, with authorship and date information if known, of the currently valid (zoological) or accepted (botanical) dwc:Taxon.
kingdom	The full scientific name of the kingdom in which the dwc:Taxon is classified.
phylum	The full scientific name of the phylum or division in which the dwc:Taxon is classified.
class	The full scientific name of the class in which the dwc:Taxon is classified.
order	The full scientific name of the order in which the dwc:Taxon is classified.
family	The full scientific name of the family in which the dwc:Taxon is classified.
genus	The full scientific name of the genus in which the dwc:Taxon is classified.
taxonRank	The taxonomic rank of the most specific name in the dwc:scientificName.
genericName	The genus part of the dwc:scientificName without authorship.
specificEpithet	The name of the first or species epithet of the dwc:scientificName.
taxonomicStatus	The status of the use of the dwc:scientificName as a label for a taxon. Requires taxonomic opinion to define the scope of a dwc:Taxon. Rules of priority then are used to define the taxonomic status of the nomenclature contained in that scope, combined with the experts opinion. It must be linked to a specific taxonomic reference that defines the concept.

**Table 2.** List of EUNIS habitats included in the dataset.

**Habitat code**	**Habitat name**
J5	Highly artificial man-made waters and associated structures.
MB2541	Mediterranean *Sabellaria alveolata* worm reefs.
MB45	Mediterranean infralittoral mixed sediment.
MB55	Mediterranean infralittoral sand.
MB553	Biocenosis of Mediterranean superficial muddy sands in sheltered waters.
MB15	Mediterranean infralittoral rock.
MB252	Biocenosis of *Posidonia oceanica*.
MA152	Biocenosis of Mediterranean pools of variable salinity.
MB151	Biocenosis of Mediterranean infralittoral algae.
MA25	Mediterranean littoral biogenic habitat.
MB151?	Facies and association of coralligenous biocenosis (in enclave).
MA2551	Vermetid reefs (*Dendropoma petraeum*).
MB35	Mediterranean infralittoral coarse sediment.
MH	Pelagic water column.
MA551	Biocenosis of Mediterranean supralittoral sands.
